# Clinical characteristics and pathogen analysis of bronchoalveolar lavage fluid in elderly patients with community‐acquired pneumonia

**DOI:** 10.1002/iid3.813

**Published:** 2023-04-27

**Authors:** Rui‐Nan Guo, Zhang Dan, Zhu Fan, Jing‐Jing Jin, Cai‐Hong Li, Bai‐Yi Liu, Xue‐Juan Li, Yan Huang

**Affiliations:** ^1^ Department of Respiratory The Affiliated Hospital of North China University of Science and Technology Tangshan China

**Keywords:** bronchoalveolar lavage fluid, community‐acquired pneumonia, elderly patients, pathogen culture

## Abstract

**Objective:**

To analyze the clinical characteristics and bronchoalveolar lavage fluid pathogens in elderly patients with community‐acquired pneumonia (CAP).

**Methods:**

This was a retrospective observational epidemiological study using that elderly cases diagnosed with community‐acquired pneumonia receiving treatment at the Affiliated Hospital of North China University of Technology, Tangshan Hongci Hospital and Tangshan Fengnan District Hospital of Traditional Chinese Medicine. A total of 92 cases were divided into two groups according to age. There were 44 patients over 75‐year‐old and 48 patients between 65 and 74‐year‐old.

**Results:**

Compared with the elderly 65 to 74‐year‐old, the elderly over 75‐year‐old with diabetes are more likely to suffer from CAP (35.42% vs. 63.64%, *p* = 0.007) and are more likely to have mixed infections (6.25% vs. 22.73%, *p* = 0.023) or larger lesions (45.83% vs. 68.18%, *p* = 0.031). Their hospital stays will also be extended (39.58% vs. 63.64%, *p* = 0.020), and the albumin level (37.51 ± 8.92 vs. 30.93 ± 6.58, *p* = 0.000), the neutrophils level (9.09(6.26–10.63) vs. 7.18(5.35–9.17),*p* = 0.026) is significantly lower and the d‐dimer (505.42 ± 197.12 vs. 611.82 ± 195.85, *p* = 0.011), PCT (0.08 ± 0.04 vs. 0.12 ± 0.07, *p* = 0.001) levels are significantly higher.

**Conclusion:**

The clinical symptoms and signs of elderly CAP patients are not so typical, and the infection is more serious. Attention should therefore be paid to elderly patients. Hypoalbuminemia and high d‐dimer can predict the prognosis of patients.

## Graphical abstract

The clinical signs of elderly community‐acquired pneumonia patients are not so typical. Attention should be paid to elderly patients. Compared with patients 65 to 74‐year‐old, patients over the age of 75 were more likely to have infections with mixed infection. They have higher PCT、D‐D and lower ALB, which indicated severe infection.

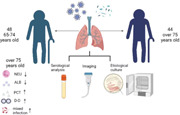

## 1 INTRODUCTION

Community‐acquired pneumonia (CAP) is a global disease. Developing countries bear a significant brunt of the disease, impacting healthcare.^1^ Many literatures show that CAP has a high incidence and hospitalization rate (11‰ and 4‰, respectively) and is more likely to recurrence.^2^, ^3^, ^4^, ^5^ With the medical interventions level gets more and more advanced and the treatment methods are constantly developing, however, the mortality rate of this disease has not decreased significantly.^6^

Population aging is a problem for countries all over the world. People aged 60 and older make up 12.3% of the global population. The number will rise to almost 22% by 2050.^7^ According to the Chinese seventh census survey, the elderly population over the age of 65 accounted for 13.50% of the general population in 2020.^8^ In China, lower respiratory tract infections are the tenth leading cause of death among the elderly population.^9^ Because of decreased organ function, an aging immune system, underlying diseases, malnutrition, and other reasons, the elderly are more easily to suffer from CAP. In addition, compared with normal adults, elderly patients with CAP have higher treatment costs and poorer clinical outcomes.^10^ Therefore, the management and prevention of susceptible diseases in the elderly are particularly important.

Bronchoalveolar lavage fluid (BALF) is a noninvasive, well‐tolerated procedure that is an important diagnostic tool for the diagnosis of various lung diseases.^11^ Metagenomic next‐generation sequencing (mNGS) of bronchoalveolar lavage fluid (BALF) has the potential to improve pathogen identification in community‐acquired pneumonia (CAP).^12^ As a new pathogen detection technology, metagenomic next‐generation sequencing (mNGS) is increasingly used in clinical practice.^13^ In many cases, culture results are the gold standard for diagnosis.^14^ Therefore, bronchoalveolar lavage fluid culture can provide pathogenic diagnosis and early treatment for patients with community‐acquired pneumonia, especially the elderly with reduced sputum production capacity.

According to these data and background, in this study, we evaluated the clinical characteristics and pathogen analysis results of the disease in the elderly patients by screening elderly patients with CAP being treated at the Affiliated Hospital of North China University of Technology, Tangshan Hongci Hospital and Tangshan Fengnan District Hospital of Traditional Chinese Medicine, providing some ideas for the management in CAP elderly patients.

## 2 METHODS

### 2.1 Design, setting, and participants

This retrospective study selected 92 elderly patients with community‐acquired pneumonia. Most patients live with their spouses or children, while a few patients live alone. All the patients were admitted to the Department of Respiratory Medicine, Affiliated Hospital of North China University of Science and Technology, Tangshan Hongci Hospital and Tangshan Fengnan District Hospital of Traditional Chinese Medicine from September 2020 to September 2021, and were divided into two groups according to age. There were 48 patients between 65 and 74‐year‐old (69.21 ± 2.52 years) and 44 patients over 75‐year‐old (78.23 ± 2.93 years). The selected patients all met the diagnostic criteria, inclusion criteria, and exclusion criteria of the Guidelines for the Diagnosis and Treatment of Community‐Acquired Pneumonia.^15^ Inclusion criteria were as follows: (1) Aged over 65 years; (2) All patients meet the diagnostic criteria of CAP; (3) All patients have complete medical records; (4) All patients underwent transnasal bronchoscopy through standard operation, and collected bronchoalveolar lavage fluid (BALF). Exclusion criteria were as follows: (1) Unclear diagnosis or incomplete data; (2) Recent severe cardiovascular and cerebrovascular diseases; (3) Severe immunodeficiency, coagulation disorder, hyperthyroidism, or malignant tumor; (4) Severe liver and kidney damage; (5) Recent severe inflammatory disease; (6) Poor compliance or coma; (7) Transfer to ICU. Inclusion criteria (1) Community onset (2) Pneumonia‐related clinical manifestations: newly developed cough, expectoration, or aggravation of existing respiratory disease symptoms with purulent sputum; with or without chest pain; fever; signs of lung consolidation and/or smell and wetness Rales; WBC > 10 × 109/L or <4 × 109/L, with or without left shift of nucleus (3) Chest X‐ray examination showed patchy, patchy infiltrative shadows or interstitial changes, with or without With pleural effusion. In line with (1) and (3), and any one of the clinical manifestations in (2) can be diagnosed.

### 2.2 Data collection

General information collected includes: gender, age, body mass index (BMI), combined underlying diseases (diabetes, hypertension, coronary heart disease, and chronic obstructive pulmonary disease [COPD]) (Table 1). Clinical symptoms include fever, respiratory symptoms (cough, expectoration, blood in sputum, and dyspnea), accompanying symptoms (anorexia, weight loss), hypokalemia, and hyponatremia. Signs: rales and the degree of lobe involvement. Laboratory indicators: results of the initial serological tests within 24 h of admission in hospital, including platelets (PLT), d‐dimer (D‐D), neutrophils (NEU), high‐sensitivity C‐reactive protein (hsCRP), serumamyloid A (SAA), fibrinogen (FIB), the erythrocyte sedimentation rate (ESR), procalcitonin (PCT), and albumin (ALB). Pathogenic detection:collect and culture of BALF.

**Table 1:** Basic characteristics of patients with CAP between two groups.

	65 to 74‐year‐old	Over 75‐year‐old	*p* Value
Gender(male,%)	29(60.41)	26(59.10)	0.588
BMI	24.12 ± 3.70	23.04 ± 4.10	0.190
Comorbidities(*n*,%)			
Diabetes	17(35.42)	28(63.64)	0.007
Coronary heart disease	8(16.67)	11(25.00)	0.324
Hypertension	17(35.42)	14(31.82)	0.715
COPD	1(2.08)	3(6.82)	0.346
Pleural effusion	18(37.50)	16(36.36)	0.910
Hypokalemia	5(10.42)	5(11.36)	1.000
Hyponatremia	13(27.08)	13(29.55)	0.793
Hospital stay(over 1 week(*n*), %)	19(39.58)	28(63.64)	0.020

Abbreviations: CAP, community‐acquired pneumonia; COPD, chronic obstructive pulmonary disease.

According to the 2017 *Chinese Expert Consensus on Pathogen Detection of Bronchoalveolar Lavage in Pulmonary Infectious Diseases*,^16^ after three injections of normal saline (about 50 mL/time) into the lesion site of the patient, BALF was recovered by negative pressure suction (all recovery rates were >30%). The obtained specimens are marked and packed into sterile containers, sent to the laboratory within 2 h at room temperature, and cultured within 24 h. The standard lavage fluid samples were inoculated on blood plates, chocolate plates, and sauropa plates for culture, and bacteria and fungi were isolated and identified by conventional methods. The serum of the patients was collected both 24 h after admission and after treatment. *Mycoplasma pneumoniae* antibodies were detected by passive agglutination method.

### 2.3 Pathogen detection positive standard

According to the expert consensus,^15^, ^16^ a BALF bacterial culture count ≥10^4^ CFU/mL (semiquantitative culture +~++) or sterile antipollution BALF ≥ 10^3^ CFU (semiquantitative culture+) is positive, and the dominant strains have moderate growth (+++) which is clinically significant; two serum *M. pneumoniae* antibody titers collected at intervals of 2 to 4 weeks showed a ≥4‐fold increase or decrease with clinical significance.

### 2.4 Statistical method

Categorical variables are shown as proportions, and continuous variables are shown as means with standard deviation (SD). The statistical tests conducted for continuous variables were the *t*‐test or Mann–Whitney U test. For categorical variables, we used the *χ* ^2^ test. *p* < 0.05 (two‐tailed) was considered statistically significant.

## 3 RESULTS

### 3.1 Basic characteristics of patients with CAP between two groups

Patients older than 75‐year‐old had a higher incidence of diabetes (*p* = 0.007) than patients 65 to 74‐year‐old. There was a statistically significant difference in the number of days in hospital between the two groups. There were no differences in age, BMI, coronary heart disease, hypertension, COPD, hypokalemia, hyponatremia, and pleural effusion between the two groups (*p* > 0.05).

### 3.2 Comparison of signs, imaging and main clinical manifestations of CAP patients between two groups

Cough (68.75% vs. 65.91%) and anorexia (58.33% vs. 84.09%) were common clinical manifestations in both groups. Fever (62.50% vs. 31.82%) was also more common in patients aged 65 to 74‐year‐old than in patients over 75‐year‐old. There were differences in fever (*p* = 0.003), dyspnea (10.42% vs. 27.27%, *p* = 0.037) and anorexia (*p* = 0.049) were identified between the two groups (*p* < 0.05), but no significant difference in other clinical manifestations was detected (*p* > 0.05). There was no significant difference in pulmonary signs between the two groups (*p* > 0.05), but pulmonary infection in the elderly over 75‐year‐old was more likely to involve more than three lung segments (45.83% vs. 68.18%, *p* = 0.031, Table 2).

**Table 2:** Comparison of signs, imaging, and main clinical manifestations of CAP patients between two groups.

	65 to 74‐year‐old	Over 75‐year‐old	*p* Value
Signs(*n*,%)			
rales in the lungs	26(54.17)	24(54.55)	0.971
Imaging (*n*,%)			
Cumulative number of lung segments (>3)	22(45.83)	30(68.18)	0.031
Clinical symptoms (*n*,%)			
Fever	30(62.50)	14(31.82)	0.003
Cough	33(68.75)	29(65.91)	0.772
Sticky phlegm	18(37.50)	11(25.00)	0.197
bloody sputum	7(14.58)	2(4.55)	0.162
Difficulty breathing	5(10.42)	12(27.27)	0.037
Chest pain	10(20.83)	6(13.64)	0.363
Loss of appetite	28(58.33)	37(84.09)	0.049
Thin	8(16.67)	13(29.55)	0.142

Abbreviation: CAP, community‐acquired pneumonia.

### 3.3 Pathogen composition ratio of all CAP patients

Among the 92 patients in this study, 57 patients had pathogens detected bronchoalveolar lavage fluid cultures or through passive agglutination tests in serum infection. The detection rate was 61.96%. The bronchial lavage fluid of all patients was stained with gram. A total of 32 patients detected Gram‐negative bacilli (13 in the younger group and 19 in the other group), and 24 patients detected Gram‐positive cocci (10 in the younger group and 14 in the other group). A total of 107 strains of 11 categories were detected. The composition ratio is shown in Table 3. Compared with patients 65 to 74‐year‐old, patients over the age of 75 were more likely to have infections with more than three pathogens (6.25% vs. 22.73%, *p* = 0.023). The number of infected strains in the two groups of patients is compared in Table 4.

**Table 3:** Pathogen composition ratio of two groups of patients.

Pathogen	Number	Comparison(%)
Gram‐positive cocci		
*Streptococcus pneumoniae*	23	21.50
*Staphylococcus aureus*	2	1.87
Gram‐negative bacilli		
Haemophilus influenzae	13	12.15
*Klebsiella pneumoniae*	12	11.21
*Pseudomonas aeruginosa*	12	11.21
*Acinetobacter baumannii*	8	7.48
*Escherichia coli*	7	6.54
Other negative bacilli	3	2.81
Fuguns/atypical pathogens		
Mycoplasma	14	13.08
*Candida albicans*	11	10.28
Aspergillus	2	1.87
Total	107	100

**Table 4:** Comparison of the number of infected strains and Gram staining results between two groups.

	65 to 74‐year‐old(*n*,%)	Over 75‐year‐old(*n*,%)	*p* Value
Infected strains			
0	20(41.67)	15(34.09)	0.455
1	11(22.92)	8(18.18)	0.575
2	14(29.16)	11(25.00)	0.654
≥3	3(6.25)	10(22.73)	0.023
Gram staining results			
Gram‐negative bacilli	13(27.08)	19(43.18)	0.383
Gram‐positive cocci	10(20.83)	14(31.82)	0.322

### 3.4 Comparison of laboratory indicators between two groups

There were significant differences between the two groups in relation to levels of NEU, ALB, D‐D, PCT (*p* = 0.026, *p* = 0.000, *p* = 0.011, *p* = 0.001), as well as other indicators. The mean levels of NEU (9.09（6.26–10.63） vs. 7.18（5.35–9.17）) and ALB (30.93 ± 6.58 vs. 37.51 ± 8.92) in patients over 75‐year‐old were lower than those 65–74 years old. The mean levels of D‐D (611.82 ± 195.85 vs. 505.42 ± 197.12) and PCT (0.12 ± 0.07 vs. 0.08 ± 0.04) were higher in patients over age of 75‐year‐old than those aged 65–74‐year‐old. There was no significant difference between the two groups in terms of levels of PLT, FIB, hsCRP, SAA, ESR, and other indicators (*p* > 0.05, Table 5).

**Table 5:** Comparison of laboratory indicators between two groups.

Laboratory Metrics	65 to 74‐year‐old	Over 75‐year‐old	*p* Value
NEU(×10^9^/L)	9.09(6.26–10.63)	7.18(5.35–9.17)	0.026
PLT(×10^9^/L)	297.15 ± 95.06	270.61 ± 88.65	0.171
ALB(g/L)	37.51 ± 8.92	30.93 ± 6.58	0.000
FIB(g/L)	5.63 ± 1.90	5.93 ± 1.85	0.456
D‐D(ng/mL)	505.42 ± 197.12	611.82 ± 195.85	0.011
PCT(ng/mL)	0.08 ± 0.04	0.12 ± 0.07	0.001
hsCRP(mg/L)	17.32(11.50–26.82)	21.99(13.51–33.96)	0.075
SAA(mg/L)	26.57(15.08–68.31)	27.66(10.64–289.67)	0.567
ESR(mm/h)	25.50(14.75–44.75)	25.00(17.00–42.00)	0.900

Abbreviations: ESR, erythrocyte sedimentation rate; NEU, neutrophils; SAA, serumamyloid A.

## 4 DISCUSSION

The elderly are susceptible to CAP due to the decline of the local defense mechanism and immune function of the respiratory system, many underlying diseases, and frequent antibiotic use.^4^, ^17^, ^18^ Thirty‐day mortality in elderly patients with CAP ranges from 1% to 25%, depending on the severity of pneumonia, comorbidities, and age.^19^, ^20^, ^21^ The clinical manifestations of CAP are usually chills, fever, cough (with or without expectoration), shortness of breath, and chest pain. However, various of reasons make elderly CAP patients' clinical features are not so obvious. They are more likely to progress to severe pneumonia, which posing a serious threat to their life.

In this study, over 75‐year‐old patients with diabetic CAP were higher than 65 to 74‐year‐old patients. Diabetes patients are more likely to have CAP and may have a worse prognosis and shorter survival.^21^, ^22^ It may be because hyperglycemia leads neutrophil and monocyte function impaired. Diabetes patients have impaired immune function and lung function, so microbial pathogens are more easily to colonization in the respiratory tract. Systemic bacterial and viral infections in CAP patients can induce stress‐induced hyperglycemia, and some pathogens may become more virulent in hyperglycemic environments, and thus have a worse prognosis.^22^, ^23^, ^24^ For older patients with diabetic CAP, it is necessary to be vigilant and intervene as soon as possible.

The main clinical manifestations of elderly CAP patients in this study were fever, cough, and loss of appetite. This is similar to the research by Huai et al.^17^ and Liu.^25^ Although there was no statistically significant difference in the clinical manifestations of coughing sticky phlegm between the two groups of CAP patients, the proportion of those was not high in both group. This is because by the increase of age, the patient's ability to cough and clear the respiratory tract becomes increasingly weaker and the sputum is too thick and difficult to cough up.^17^ Therefore, the recovery of elderly CAP patients is slower than that of normal adults, and the cost is higher. In our study, the number of people over 75‐year‐old with dyspnea is relatively high, and the number of patients with fever is significantly less than that of patients 64–75‐year‐old group. In addition, patients of CAP aged over 75‐year‐old was more likely to involve more than three lobes. It's possible that due to the higher incidence of diabetes, the patients in this group have impaired immunity, pulmonary host defense disorders, and pulmonary inflammatory dysfunction^2^; it is also possible that this group is older and has a weaker immune response, so the infection more serious.^25^ Some studies have shown that pneumonia with multiple‐lobes infection is a risk factor for poor prognosis.^23^ Therefore, when treating elderly CAP patients, it is necessary to realize that the clinical manifestations of such patients are not typical. Strengthening medical awareness of patients and their families and seeking medical attention as soon as possible are the keys to preventing the progression of the disease.

According to several literatures, the most common pathogen of CAP is the *influenza virus*, followed by *Streptococcus pneumoniae*. Other pathogens include but are not limited to *Haemophilus influenzae*, *Pseudomonas aeruginosa*, *Klebsiella pneumoniae*, *Escherichia coli*, *Staphylococcus aureus*, *Chlamydia pneumoniae*, *M. pneumoniae*, *Legionella pneumophila*, *Moraxella catarrhalis*, and so forth.^20^, ^26^, ^27^ Our study investigated the culture results of lavage fluid from elderly patients with CAP. The top three pathogens were *S. pneumoniae* (21.50%), *M. pneumoniae* (13.08%), and *H. influenzae* (12.15%). At the same time, the number of patients with mixed infection in the two groups is large. People who regularly take antibiotics have immune disorders. They are more easily to meaning that they produce drug‐resistant strains, thus increasing the chance of infection with multiple pathogens.^17^ Most of the patients with mixed infection were accompanied by gram‐negative bacteria, which may be related to comorbidities or the contamination of specimens during the patient's hospital stay.^20^ The increasing resistance of Gram‐negative bacteria to carbapenems and limited treatment options increase the risk of death for these patients.^28^, ^29^ In this study, patients over 75 years of age were more likely to have 3 strains than patients 65 to 74‐year‐old, and the number of co‐infected patients was also greater than that of patients 65 to 74‐year‐old. Therefore, it is necessary to intervene in medication as soon as possible. Drug susceptibility tests should be supplied if necessary, so as to improve the prognosis of patients as much as possible. In addition, because *M. pneumoniae* infection is also more common. Therefore, it is necessary to cover this pathogen when taking medicine. The literature shows that drug‐resistant Gram‐negative bacilli mainly include Acinetobacter baumannii, *K. pneumoniae*, ESBL‐producing Enterobacterales and Pseudomonas aeruginosa.^30^, ^31^, ^32^ In particular, carbapenem‐resistant gram‐negative bacilli (CR‐GNB) have become a global threat. Because they are highly resistant to most antibiotics.^33^ In this study, more than half of the CAP patients had these bacteria in their etiological cultures. This means that the use of antibiotics in these groups is limited. According to the literature, generally these bacteria are sensitive to meropenem, imipenem, colistin, and so forth.^34^, ^35^, ^36^ However, due to time problems, the variable antibiotics could not be collected in this experiment. We will make improvements in follow‐up research. In addition, there is no difference in Gram staining results of bronchial lavage fluid between the two groups, which may be caused by the small sample size, and the sample size may be expanded for further exploration.

It has been^24^ reported that important immune cells, such as neutrophils and lymphocytes are elevated during infection and are positively correlated with patient immunity. In this study, the increase in the number of neutrophils in the elderly patients over 75‐year‐old was lower than in the patients 65 to 74‐year‐old, suggesting that the immunity of older patients was worse. At the same time, the serum albumin content of this group of patients was significantly lower than that of patients 65 to 74‐year‐old. Several studies^37^, ^38^, ^39^, ^40^ have shown that as an indicator of malnutrition, hypoalbuminemia is associated with disease severity in CAP patients. The albumin level at admission affects patient prognosis and mortality. It also has been reported that high levels of procalcitonin are associated with poor prognosis in elderly patients, and patients with elevated procalcitonin have a lower survival rate.^20^, ^40^ However, the literature also proposes that procalcitonin level is only associated with mortality in patients under 74 years of age, it cannot be used as a risk factor for mortality in patients with CAP over 75 years of age.^20^ In our study, the procalcitonin levels of the two groups of patients were significantly different, and the CAP patients over 75‐year‐old were significantly higher than those of 64 to 75‐year‐old patients. However, considering that the group of patients over 75‐year‐old is mostly complicated with diabetes, some studies have shown that the optimal point of PCT detection^41^ in such patients is higher than that of normal people. Therefore, more experiments are needed to explore the exact relationship between procalcitonin and the prognosis of elderly CAP patients. At the same time, there was no significant difference in hsCRP between the two groups of patients in this study. However, according to multiple literature reports,^10^, ^42^, ^43^ high levels of CRP are indeed related to patient mortality and prognosis. Combined with other clinical indicators and imaging results, this experiment may need to expand the sample size.

When the body is in a state of infection, platelets will decrease. In the American CAP guidelines, thrombocytopenia is also listed as one of the diagnostic criteria for severe CAP.^44^, ^45^ However, in this study, no difference was observed in the number of platelets between the two groups, and there was no significant decrease in platelets between the two groups. This may be caused by insufficient sample size. The contents of D‐D and FIB in both groups were over normal values, and D‐D and FIB contents in patients with CAP over 75‐year‐old were significantly higher than those in patients aged 65–74 years. Meta‐analyses by Li et al.^46^ and Yang et al.^47^ showed that the level of d‐dimer in patients with severe CAP was significantly higher than that in patients with nonsevere CAP, and D‐D was considered to have extremely high clinical value, which could be used as a means of judging the prognosis of patients degree indicator. This is consistent with the analysis results of our study.

This study also has shortcomings. Due to limited equipment and conditions, virus isolation and detection in patient lavage fluid was not performed; and the sample size needs to be further expanded.

## 5 CONCLUSION

In conclusion, clinical manifestations are not typical in patients with CAP over 75‐year‐old. They are usually serious, and mostly have a mixed infection. They usually have longer hospital stays and are more difficult to treat, and the prognosis may not be ideal. Therefore, attention should be paid to the elderly group and prevention should be strengthened. It is an important measure to improve the knowledge of such people and their families about related diseases. ALB and D‐D can predict the prognosis of patients to a certain extent.

## AUTHOR CONTRIBUTIONS

*Conception and design of the research*: Yan Huang. *Acquisition of data*: Cai‐Hong Li. *Analysis and interpretation of the data*: Bai‐Yi Liu. *Statistical analysis*: Dan Zhang, Zhu Fan, and Jing‐Jing Jin. *Obtaining financing*: Xue‐Juan Li. *Writing of the manuscript*: Rui‐Nan Guo. *Critical revision of the manuscript for intellectual content*: Yan Huang. All authors read and approved the final draft.

## CONFLICT OF INTEREST STATEMENT

The authors declare no conflict of interest.

## ETHICS STATEMENT

This study was conducted with the approval of the Affiliated Hospital of North China University of Science and Technology Ethic Committee. This study was conducted in accordance with the declaration of Helsinki. Written informed consent was obtained from all participants.

## Data Availability

The data sets used and/or analyzed during the current study available from the corresponding author on reasonable request.
